# Evaluation of patients undergoing placement of zygomatic implants using sinus slot technique

**DOI:** 10.1186/s40729-015-0035-x

**Published:** 2016-01-13

**Authors:** P. P. T. Araújo, S. A. Sousa, V. B. S. Diniz, P. P. Gomes, J. S. P. da Silva, A. R. Germano

**Affiliations:** Department of Oral and Maxillofacial Surgery, Hospital Universitário Onofre, Federal University of Rio Grande do Norte, Av. Nilo Peçanha, 620 - Petrópolis, Natal, RN 59012-300 Brazil

**Keywords:** Zygomatic implant, Dental implants, Edentulous maxilla

## Abstract

**Background:**

This study aimed to evaluate patients undergoing placement of zygomatic implants by Stella and Warner’s technique, considering the survival rate of conventional and zygomatic implants, and assess the health of the maxillary sinuses and the level of patient satisfaction.

**Methods:**

In this retrospective cohort study, 28 patients had received a combination of conventional and zygomatic implants (group I) and 14 were rehabilitated with only conventional implants (group II).

**Results:**

The results showed that Stella and Warner’s technique, thought to minimize the presence of the implant into the maxillary sinus, improving the emergence of the implant, proved to be effective, allowing a high survival rate of conventional and zygomatic implants (100 %). The follow-up period ranged from a minimum of 15 months to a maximum of 53 months after prosthetic rehabilitation (average of 34 months). No pathological changes were found on the periimplant tissues. Radiographs showed satisfactory bone levels in conventional implants of oral rehabilitation with zygomatic implants and a good positioning of the apex of the zygomatic implants in relation to the zygomatic bone. The tomographic findings revealed no characteristics of sinus disease. There were no cases of obstruction of the maxillary sinus ostium.

**Conclusions:**

The placement of zygomatic implants by Stella and Warner’s technique proved to be a predictable technique with high implant survival rate in patients with atrophic maxilla and was not associated with sinus disease in the sample analyzed. However, a long-term follow-up is necessary to confirm the initial findings of this study.

## Background

The prosthodontic rehabilitation of patients with atrophic maxilla is a challenge for a clinician due to the severe compromise of masticatory function and speech with a significant quality of life impact. The poor bone volume found on these patients makes it difficult for conventional rehabilitation with fixed prosthesis and to insert dental implants [1].

Different surgical techniques with varying degrees of success rate have been described in the literature to deal with cases of maxillary atrophy. Techniques such as major reconstructions using bone graft from the iliac crest associated or not with Le Fort I osteotomy are the most common ones used for these cases. However, these techniques have important biological cost requiring long periods of treatment and are more sensitive to technical errors [2, 3]. The morbidity of these techniques includes the possibility of sinusitis, neurosensory disorders, contamination or exposure of the graft, postoperative pain, mobility difficulties, and insufficient remanent bone after the healing period [4].

The emergence of the zygomatic implants from Brånemark’s [5] studies gave the surgeons the possibility to obtain a firm anchorage of implants to the zygomatic bone, making the rehabilitation of an atrophic maxilla possible with two or four implants in the anterior maxilla [6]. The high success rate shown by the first protocol suggested by Brånemark [5] triggered a series of studies and the publication of different modifications of the guidelines for zygomatic implants [7–9].

**Fig. 1:**
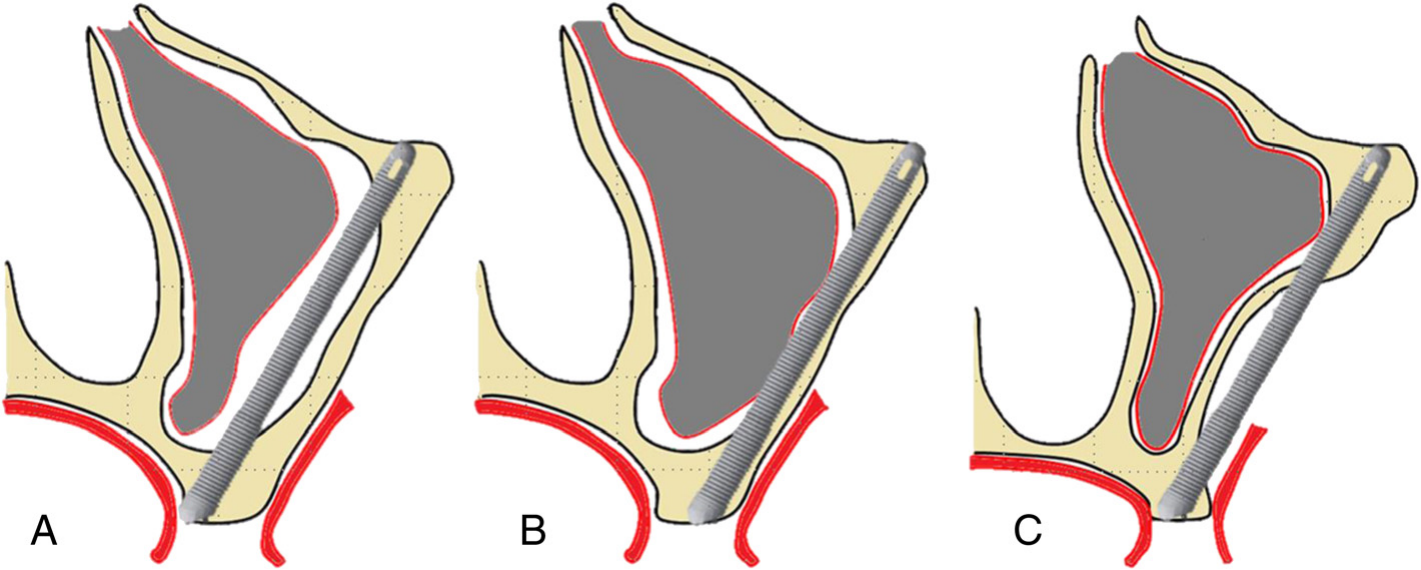
**a** Brånemark technique. **b** Sinus slot technique. **c** Extrasinus technique

The original protocol performed by Brånemark [5] involved the opening of a window at the upper side of the anterior wall of the maxillary sinus to guide the perforations. The implant is placed in an intrasinus position without elevation of the sinus membrane. This step was later modified with an elevation of the membrane to provide the retraction of the sinus mucosa that is preserved in this technique. The zygomatic implants are anchored in the upper second premolar position, passing through the maxillary sinus and making new grounding in the body of the zygoma. In this situation, the emergence of the implants mostly is located palatal to the alveolar crest (Fig. 1a). Trying to simplify this technique, Stella and Warner [10] proposed the preparation of a groove orientation on the zygomatic buttress region, extending from the base of the zygoma, approximately 5 mm of the alveolar bone crest. With this technique (sinus slot technique), the detachment of the sinus membrane is not necessary and part of the zygomatic implant is directly in the maxillary sinus. However, the implant ends up emerging on the alveolar crest level of the first molar in a more vertical angulation, which favors the interface with the prosthesis and also simplifies the placement of the implants [11] (Fig. 1b). Another technique of zygomatic implants is the extrasinus, where the implant is completely out of the maxillary sinus (Fig. 1c).

A number of papers have reported cases of maxillary sinusitis following the placement of zygomatic implants [3–17]. In a systematic review of retrospective and prospective studies, Chrcanovic and Abreu (2013) [18] found that the most common complication was maxillary sinusitis, affecting 70 cases of the 2402 zygomatic implants installed.

Many studies [5–7, 12] were conducted to evaluate the success rate of zygomatic implants for the Brånemark’s technique. But Stella and Warner’s technique still needs investigation. In this sense, the present study aimed to evaluate the success of zygomatic implants placed using this technique, investigating the survival rate of the implants, and assess the possible association between sinus disease and the placement of zygomatic implants using this technique and the satisfaction of patients rehabilitated with full fixed prostheses with zygomatic implants.

## Methods

This retrospective cohort study was submitted and approved by the Hospital Research Ethics Committee, receiving the registration number 137/201.

The sample consisted of 28 patients (21 females and 7 males), with age ranging from 46 to 63 years, and all of them have undergone either the placement of zygomatic implants using the Stella and Warner’s technique or conventional implants, in the period from 2007 to 2014 at the Oral-Maxillofacial Surgery and Traumatology Sector of the Onofre Lopes University Hospital. The patients were divided into two groups: group I comprised 14 patients who underwent surgery for installation of zygomatic implants by the Stella and Warner’s technique, rehabilitated with implant-supported fixed dentures, and group II consisting of 14 patients who were rehabilitated with total implant-supported fixed prosthesis, using conventional implants only, without the need of zygomatic implants. All patients were rehabilitated with Conexão® implants system.

The inclusion criteria for group I were patients with severe maxillary resorption, classified as classes IV and V of Cawood and Howell (1988)[19], receiving zygomatic implants using Stella and Warner’s technique, performed by the Oral and Maxillofacial Surgery Department from the Rio Grande do Norte Federal University, and having full implant-supported fixed prosthesis with at least one zygomatic implant under functional loading for at least 6 months. For the group II, individuals rehabilitated with full fixed implant-supported prosthesis without the presence of zygomatic implants, without reconstructive surgeries, and also on functional loading for at least 6 months were included. The healing time for all zygomatic implants was, at least, 6 months.

The evaluation was performed in four stages: the first was characterized by a radiographic analysis of implants in group I, the second was a clinical evaluation of implants placed in group I, the third was an evaluation of the maxillary sinus health in group I, and the fourth consisted of an application of a questionnaire to assess the degree of satisfaction of the prosthetic rehabilitation in groups I and II. The aim of this study was to evaluate the success rate of zygomatic implants using Stella and Warner’s technique [10], investigating the survival rate, sinus disease, and the satisfaction of patients rehabilitated with full fixed prostheses with zygomatic implants.

The hypothesis of this study was to analyze if Stella and Warner’s technique have high survival rates and their rehabilitation have similar satisfaction when compared to total fixed prostheses with conventional implants.

### Radiographic evaluation

**Fig. 2:**
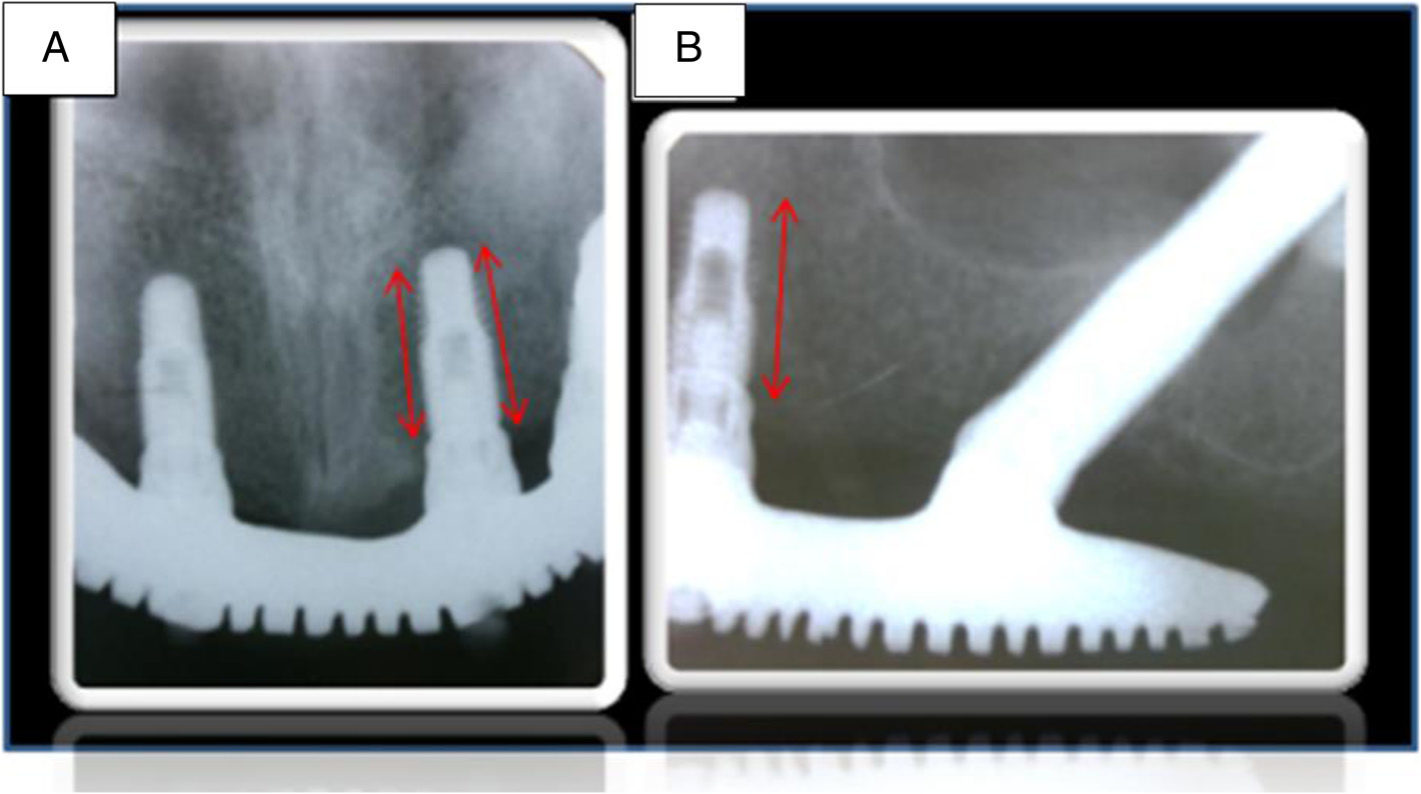
Periapical radiographs using the parallelism technique. **a** Conventional implants—anterior. **b** Conventional implants—posterior

**Fig. 3:**
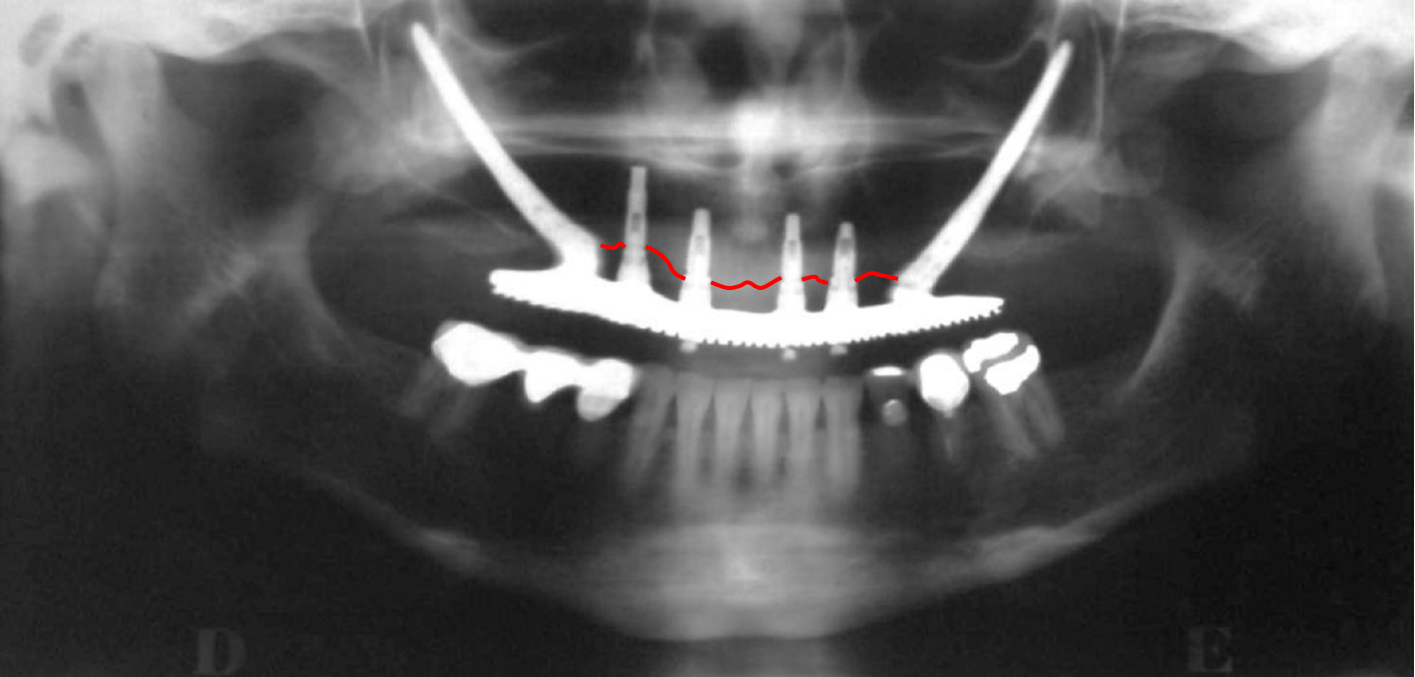
Panoramic radiograph showing bone level maintenance around the conventional implants

**Fig. 4:**
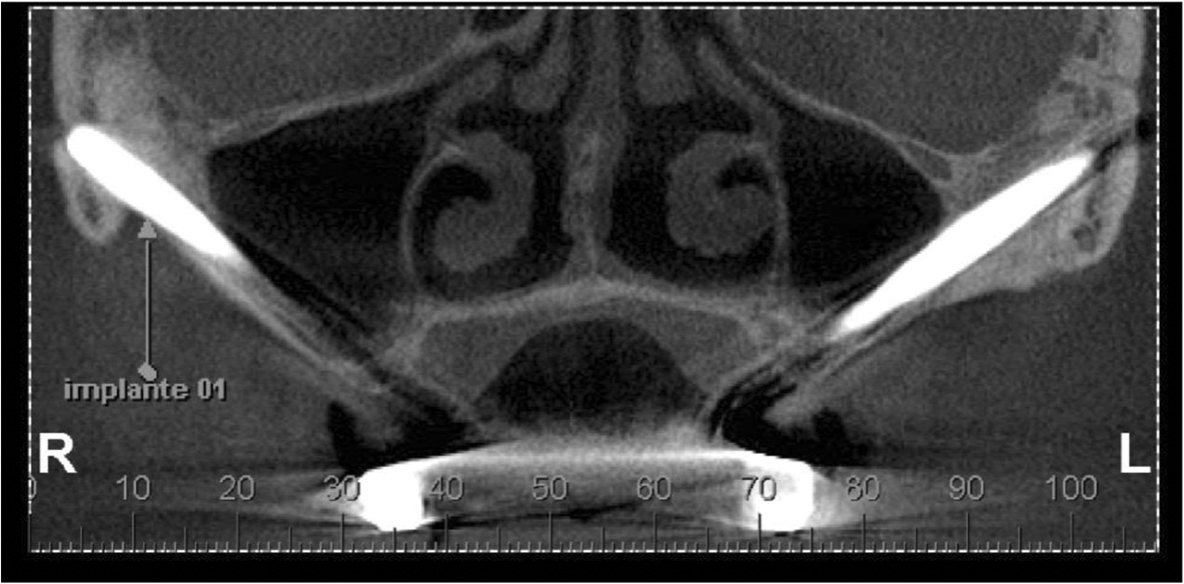
Coronal slice from the CBCT showing implant apical third inside the zygomatic bone

**Fig. 5:**
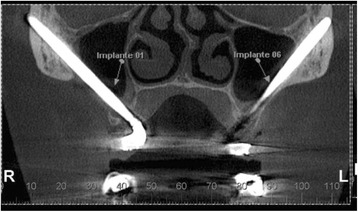
Coronal slice from the CBCT showing small exteriorization of a zygomatic implant apex

Panoramic and periapical radiographs were obtained for conventional and zygomatic implants in group I (Figs. 2a, b and 3). The purpose was to evaluate the bone level for the conventional implants, considering that up to two thirds of the total length of the implant would be acceptable as a standard of osteointegration [13]. A ruler template with the respective magnifications was used, considering 25 to 0 % for panoramic and periapical radiographs, respectively. Additionally, the radiographic criteria recommended by Buser et al. [19] were included to determine the success of the implants. These criteria consist of the absence of persistent radiolucency around the implant. The zygomatic implants were assessed only to verify their correct position, with acceptable maintenance of the apical third of the implant inside the zygomatic bone or small apical exteriorizations that did not exceed 1 mm, evaluated by a cone-beam CT scan (Figs. 4 and 5). A single and calibrated investigator collected the data in two different occasions.

### Clinical evaluation

**Fig. 6:**
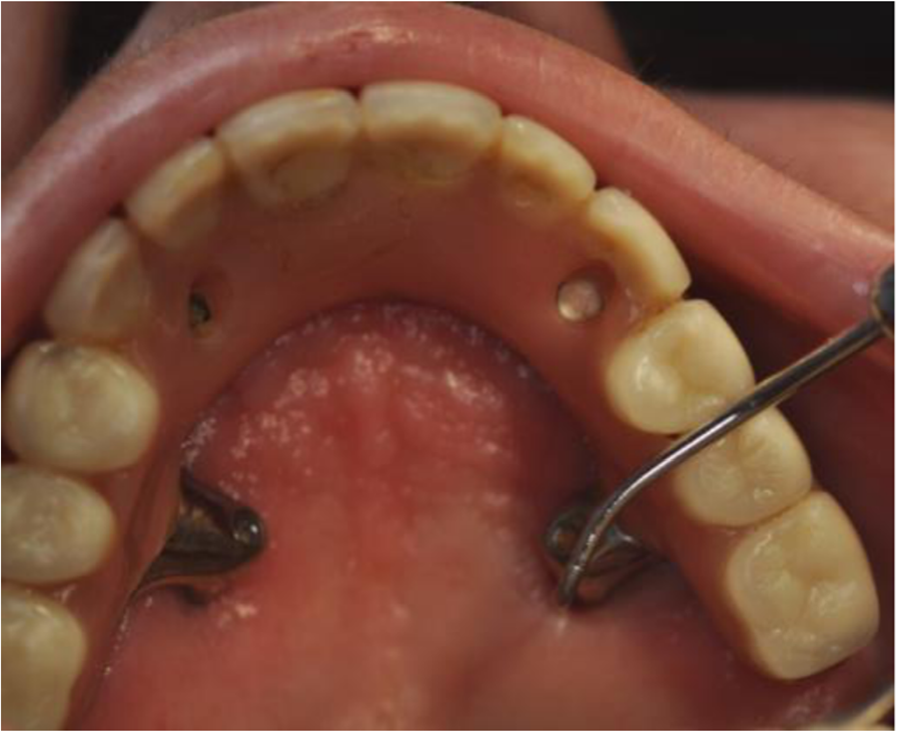
Zygomatic implant probing using a WHO periodontal probe

Group I patients went through full intraoral and health history examination with the purpose of analyzing the periimplant health. The data collected included spontaneous pain or pain on palpation; mucosa color; and presence of purulent secretion, presence of mobility [19], and presence of oral-antral fistula in the area of the zygomatic implants. The data were also supplemented probing the zygomatic implants using a WHO periodontal probe (Fig. 6). The probing examination took into consideration three parameters:

- Bleeding on probing: the probe was inserted with light pressure, avoiding overextension into the healthy tissues in the mesial, distal, buccal, and palatal implant surfaces. In cases where the probing was negative for bleeding, the periimplant site was considered healthy and stable.
- Probing depth: probing the periimplant sulcus was done with slight pressure. In cases of healthy mucosa or mucositis, the probing depth should not be greater than 5.0 mm.
- Presence of purulent secretion on probing.

### Evaluation of the maxillary sinus health

The evaluation of the maxillary sinus health was performed by a single otolaryngologist at the hospital. The clinical exam also included questions on signs and symptoms of sinus disease. Cone-beam computed tomography was performed on all patients. For patients with signs indicating maxillary sinusitis, a quality of life questionnaire was administered and video-assisted nasal fibroscopy was performed.

The aim of the clinical exam was to investigate signs of sinusitis: (a) nasal obstruction, using a visual analog scale (VAS) ranging from 0 to 10 points, on which the patient marked his/her degree of obstruction, with the examiner’s subsequent delineation of mild, moderate, or intense obstruction based on the patient’s mark; (b) periorbital edema with or without hyperemia; (c) halitosis, which could be caused by purulent secretions from the nasal fossae; and (d) facial pain (spontaneous or upon palpation) in the region of the maxillary sinuses.

Cone-beam computed tomography (I-CAT™) was used for the analysis of the maxillary sinus (coronal and axial cuts of 1.0 mm in thickness). Panoramic reconstruction was used to determine the presence of opacification of the sinus (Fig. 2) and obstruction of the ostium (Fig. 4). The Lund-McKay [20] scoring system was used for the assessment of maxillary sinus abnormalities on the tomograms: 0 = absence of abnormalities; 1 = partial opacification; and 2 = complete opacification.

In cases of findings suggestive of maxillary sinusitis, video-assisted nasal nasofibroscopy (Fig. 5) was performed to determine the presence of edema and hyperemia of the nasal conchae, secretion in the region of the middle meatus with posterior drainage, and obstruction of the ostium. Moreover, a disease-specific questionnaire [20] was administered to those with signs suggestive of maxillary sinusitis for complementary information to make the differential diagnosis.

### Patient satisfaction rate after oral rehabilitation

**Fig. 7:**
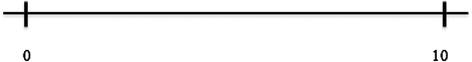
Visual analog scale—patient version

**Fig. 8:**
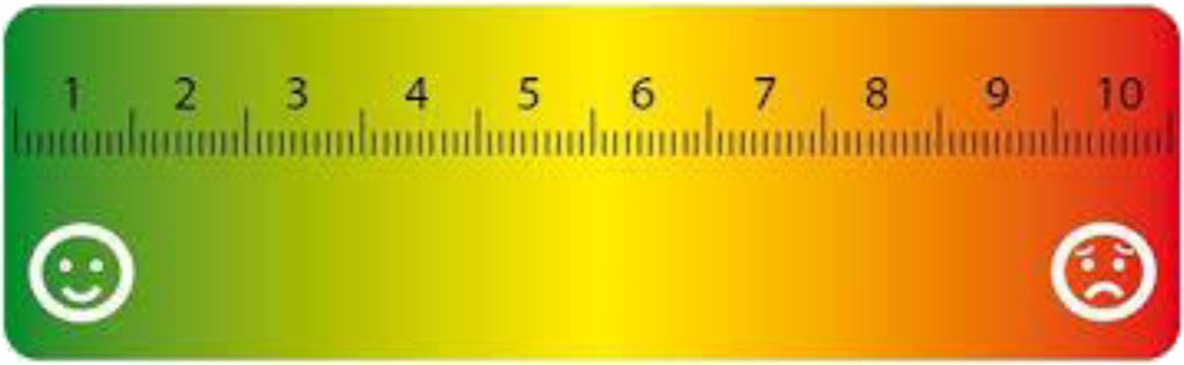
Visual analog scale—evaluator version

In the last stage of this study, the degree of satisfaction after oral rehabilitation, comparing patients rehabilitated with fixed prosthesis over zygomatic implants with fixed prosthesis supported by conventional implants only was estimated. A VAS was used, considering the stability, comfort, ability to speak, cleanability, aesthetic, self-esteem, and function as parameters (Figs. 7 and 8). Patients answered questions giving values from 0 (completely dissatisfied) to 10 (completely satisfied) for each item.

### Result analysis

The first, second, and third stages of the study were presented descriptively. The data of the fourth stage were statistically evaluated to detect the differences in the degree of satisfaction of patients rehabilitated with a combination of conventional and zygomatic implants compared with those rehabilitated only with conventional implants. The data were entered into Excel spreadsheets (Microsoft ® Office XP Professional), exported to DBF format (Data Base Format), and subsequently analyzed and statistically treated by (Statistical Package for the Social Sciences version 1.7 for Windows XP ® SPSS; Chicago, IL, USA). *T* test for independent samples, considering the level of significance of 5 % (*p* < 0.05), was used.

## Results

Of the 17 operated patients, 14 were included in the study and 2 were excluded for not having enough data in the chart and 1 for refusing to return for evaluation of sinus health, totalizing 27 zygomatic implants and 55 conventional implants in group I, without losing any implant, representing 100 % survival of implants placed. The minimum follow-up was 15 and the maximum was 53 months (average of 34 months) after installation of fixed denture for group I. The emergence of the implants occurred between the regions of the second premolar and first molar. In class V cases of Cawood and Howell (1998), the emergence was more palatinized.

### Implants radiographic evaluation

The bone level observed in conventional implants appeared to be above two thirds of their total length in 96.3 % of the cases, with only 3.6 % having the bone level at the two-third mark. Most cases of bone loss were in the posterior region. No cases of persistent radiolucency around the implants were observed.

The zygomatic implants showed that they were adequately positioned with the apical third into the bone, with externalization of 1 mm in 22.2 % of the cases.

### Clinical evaluation

Pain or purulent secretion on palpation was not observed to be associated with any of the inserted implants either conventional or zygomatic. The color of the mucosa was normal in 100 % of installed conventional implants and in 96.29 % of zygomatic implants, with only one implant showing redness in the palate on all sides of the implant.

The probing of conventional implants was not possible to perform due to the length of the prosthesis, as they were not removed in the study. Only the zygomatic implants could be probed to analyze the periimplant sulcus. There was no purulent discharge or bleeding in any of the surveyed sites. The probing depth ranged from 2 to 3 mm in all surfaces, showing the absence of inflammatory pathologies and appropriate levels of depth.

### Evaluation of the maxillary sinus health

Among the 14 patients submitted to zygomatic implants, only three reported having had nasal obstruction in the weeks preceding the evaluation. One of these patients had a cold, and the other two reported having self-medicated with antihistamines 1 week prior to the evaluation, with the subsequent disappearance of nasal obstruction. Only one patient reported headache and the sensation of pressure in the region of the maxillary sinus. This patient was submitted to video-assisted nasal fibroscopy, which revealed the maintenance of ostium patency and no drainage of secretions into the middle meatus (bilaterally). The tomographic exam demonstrated the absence of sinusitis. No patient had pain upon palpation or periorbital edema. Only one patient exhibited halitosis, but this clinical sign was related to poor oral hygiene rather than sinusitis.

The tomographies revealed no obstruction of the maxillary ostium in any patient and all implants were in close contact with the maxillary sinus (partially inside the sinus cavity) (Fig. 5). All maxillary sinuses received grade 0 on the Lund-McKay scoring system [21], demonstrating an absence of abnormalities in the images (Table 1).

**Table 1:** Statistical analysis of individual parameters

	Zygomatic implant group				Conventional implant group				
Variable	*N*	Mean	PD	IC 95 %	*N*	Mean	PD	IC 95 %	*p*
Overall satisfaction	14	8.88	0.71	−1.17/−0.38	14	9.65	0.13	−1.19/−0.36	0.001
Stability	14	9.79	0.54	−0.50/0.09	14	10.00	0.00	−0.52/0.11	0.009
Ease of cleaning	14	5.82	1.99	−3.49/−1.14	14	8.15	0.78	−3.52/−1.11	0.045
Ability to speak	14	8.78	1.73	−2.16/−0.26	14	10.00	0.00	−2.21/−0.21	0.00
Aesthetics	14	9.64	0.90	−0.73/0.32	14	9.85	0.32	−0.75/0.33	0.05
Self-esteem	14	9.66	0.85	−0.80/0.13	14	10.00	0.00	−0.82/0.15	0.002
Masticatory function	14	9.52	1.08	−1.03/0.16	14	9,95	0,16	−1.06/0.19	0.003

### Patient satisfaction rate after oral rehabilitation

Considering the hypothesis that the palatal emergency profile of zygomatic implants determines a less satisfactory prosthetic rehabilitation, we compared the degree of patient satisfaction with group II. Applying the *t* test for independent samples, statistically significant difference between the group rehabilitated with zygomatic implants and the group rehabilitated without them was verified. This finding was noticed in the overall patient satisfaction and also individually for each variable. Individual parameters included the stability of the prosthesis, ease of cleaning, the ability to speak, aesthetics, self-esteem, and masticatory function after prosthetic rehabilitation (Table 1).

## Discussion

The morbidity caused by bone graft harvesting and the delay in the final treatment due to the time necessary for bone incorporation triggered the development of techniques without grafting as an option for the treatment of patients with edentulous jaws [8]. Brånemark in 1998 developed a novel technique for placing implants in the zygomatic bone to treat severely atrophic maxilla without the need for grafting, which was later modified by Stella and Warner [10]. The later minimized the presence of the implant into the maxillary sinus, improving the emergence of the implant, since it allowed a more vertical angle than the original technique. Many prospective and retrospective studies [1, 5, 6, 12, 14–17, 22–24] showed good results by using the original technique, while only few researches [11, 18] discuss Stella and Warner’s technique. So, this retrospective study aimed to evaluate Stella and Warner’s technique, contributing to a greater scientific validation.

Fourteen patients who underwent placement of zygomatic implants were evaluated over a period ranging from 15 to 53 months, where 100 % survival rate of conventional and zygomatic implants involved in the rehabilitation was observed. This represented a survival rate compatible with Brånemark’s studies [14, 19] that showed a survival rate for zygomatic implants of 94.2 to 100 % after 5 to 10 years and 12 years of follow-up, respectively. Different authors [1, 6, 11, 15–18, 21, 22, 24–26] reported a survival rate for these implants between 96 and 100 %. For conventional implants, some studies [11, 15, 16, 18, 21, 22] reported a survival rate ranging between 95 and 100 % [6]. The findings of our study demonstrated that the technique of Stella and Warner is fairly predictable with survival and success rates compatible with the ones described in the literature, independently on the technique used.

The bone level after loading the implants was one of the criteria used in this study to assess the survival rate of conventional implants involved in the rehabilitation. As described by Hirsch et al. [6], Aparicio et al. [15], and Farzad et al. [16], this criteria was assessed by periapical radiographs obtained by the parallelism technique combined with panoramic radiographs for conventional implants and CT scans for zygomatic implants. The bone loss was defined as a vertical change of bone level measured from the most inferior line of implant exposure. All previous studies have demonstrated satisfactory sustained levels over a period of 60 months of the load application.

One aspect to be considered is that our research is a retrospective cohort study, making it difficult to find a standardization of radiographs that could accurately determine the annual bone loss as described by Farzad et al. [16], especially for conventional implants. Sometimes when the conventional implant is slightly tilted to the palate in cases of anterior maxillary atrophy, it is difficult to obtain adequate periapical radiographs by the parallelism technique. Therefore, the methodology suggested by Lang and Lindhe [13] was used with reference to the implant bone level that should not be less than two thirds of its total length in order to have a satisfactory osseointegration. Additionally, the radiographic criteria recommended by Buser were used to analyze the presence/absence of persistent radiolucency around the implant.

For greater accuracy to assess implant osseointegration, percussion and immobility tests are described in the literature [15, 16]. Performing these tests requires the removal of fixed prosthesis, which in our view, would be justified only in cases of necessity for prosthesis replacement or when mismatches or gaps was observed in the rehabilitation and/or implants, since the osseointegration loss can also be verified by signs indicating decreased bone volume around the implant, periimplant radiolucency, the presence of spontaneous pain on palpation, local redness, and the presence of purulent secretion [6, 26, 27]. According to Von Krammer [27], periimplantar mobility is generally associated with periimplant radiolucency and this monitoring method has the advantage of not requiring removal of the prosthesis during the evaluation.

Our study demonstrated the absence of pain as well as of pus or bleeding on probing and palpation for both zygomatic and conventional implants, with good bone level for conventional implants. No periimplant radiolucency was noted around the conventional implants and in the apical portion of the zygomatic implants. These findings are similar to the studies of Stiévenart and Malevez [1], Peñarrocha et al. [18], and Davo et al. [22].

Some authors [6, 16, 17] reported inflammation of the soft tissue around the implants, being mucosa redness one of the signs. Farzad et al. [16] reported some degree of inflammation found in 14 of 22 zygomatic implants installed by the original technique. The soft tissue found around the implant appeared to be susceptible to infection justified by the authors by the increase in the number of patients with problems in performing a correct oral hygiene. In our study, only one patient had redness in the palatal region of the left zygomatic, being also associated with poor hygiene. In our opinion, probably a factor that contributes to the health of periimplant zygomatic implants is a sulcus depth within an acceptable range, which is able to promote self-cleaning by the patient during brushing and/or gum massage.

The probing depth in the palatal mucosa of zygomatic implant can be considered normal up to 5 mm, consisting of parakeratinized epithelium which is not comparable to the normal depth of the sulcus around a conventional implant [1]. In this study, both the zygomatic and conventional implants had a mean probing depth within normal limits, ranging from 2 to 3 mm, which is considered satisfactory. The technique of Stella and Warner allows a more vertical emergency profile favoring a less deep sulcus due to a more open angle obtained. Other studies have reported the presence of problems with oral tissue in the region of the zygomatic implants, including infection and swelling, usually associated with loss of implant apical osseointegration [6, 17, 28]. Hirsch et al. [6] have reported the presence of hyperplasia, mucositis, and infection in eight patients in a total of ten throughout the monitoring period.

Although certain criteria to evaluate osseointegration were considered, the study used the concept of survival of the implants instead of the success. Survival is a more general term, considering only the implant is still in the oral cavity, without analyzing the quality of its function and maintenance of its support. The success rate is applied to those fitting into established and applied criteria of installed implant [29]. Due to various factors, from the type of test, as well as the difficulty to access all implants and characteristics inherent when working with atrophic maxilla without reconstruction, we found that survival, which is a concept widely used in the literature, would fit best for this research.

The possibility of zygomatic implants causing or favoring sinus disease due to the exposure of the implants within the maxillary sinus is an important issue. A number of studies describe the occurrence of maxillary sinusitis in patients with zygomatic implants [4, 6, 8, 10–12, 15, 17, 18, 25, 26]. This finding has been attributed to perforation of the sinus membrane [17, 26], a lack of contact between the implant and the surrounding bone crest [8], the migration of bacteria from the oral cavity to the maxillary sinus due to communication between these structures [7, 17], and preexisting sinus conditions from the clinical and radiographic standpoint [4, 5, 7, 8, 10, 27].

According to Stiévenart and Malevez [1], the incidence of sinusitis ranges from 14 to 30 %. In a previous retrospective study involving patients submitted to the Stella and Warner’s technique, Peñarrocha et al. [18] found two cases of sinusitis among the 42 implants; one case was treated with antibiotics and the other was submitted to the removal of the implant. Becktor et al. [7] report that patients with oral-sinus communication may develop suppuration with or without sinusitis. In such cases, treatment consists of the administration of antibiotics and/or the repositioning of the soft tissue and maintenance of a stable zygomatic implant, with no reports of the recurrence of sinusitis [6, 14, 25]. Brånemark [5] found fistula in five patients both before and after the connection of the abutment in 1 year of follow-up. Three patients exhibited specific symptoms of sinusitis, such as nighttime pain, unilateral pain in bad weather, and obstruction of the sinus. The existence of a small amount of residual bone in the alveolar crest associated with an implant placement technique with minor destruction of the sinus region can determine a more favorable prognosis for these complications.

The risk of the development of maxillary sinusitis associated with zygomatic implants installed using the original technique is reported to be low to moderate [30]. Few data have been published regarding this risk in relation to the Stella and Warner’s technique. According to Peñarrocha et al. [18], the small slot in the zygomatic-maxillary region diminishes the likelihood of maxillary sinusitis, reporting a 4.7 % rate of occurrence of this complication in 12 months of follow-up. In the present study, the implants had two position patterns: those that partially invaded the maxillary sinus and those that were positioned alongside but completely outside the sinus due to the anatomy of the zygomatic-maxillary region encountered. However, the slot that characterizes this technique was made in both cases. The clinical and imaging findings demonstrated no cases of maxillary sinusitis in the follow-up.

According to the Brazilian Guidelines for Sinusitis [20], the clinical exam has sensitivity and specificity of 69 and 79 %, respectively, which makes the use of complementary diagnostic tools necessary. A number of authors report the use of computed tomography for the diagnosis of sinusitis. Nakai et al. [31] performed this exam 6 months following the placement of 15 zygomatic implants in nine patients and found an absence of signs and symptoms of sinusitis. Maló et al. [24] evaluated the association between zygomatic implants and maxillary sinusitis using sinusoscopy on 14 patients and found no cases of infection or inflammation of the mucosa surrounding the implants, demonstrating that titanium implants are compatible with the health and normal function of the maxillary sinus. However, the studies cited employed the original technique.

In a systematic review, Chrcanovic and Abreu [18] report that immobility of zygomatic implants is one of the main factors contributing to the homeostasis of the maxillary sinus. This immobility is accomplished by adequate anchorage of the implant in the zygomatic bone and, when possible, the maxillary bone as well as a firm connection with the overdentures. The rigorous selection of patients with no history of active sinus disease is another important factor and was confirmed in the present sample through preoperative computed tomography, following the normal routine of the hospital at which this study was carried out.

Computed tomography is currently the method of choice for the determination of sinusitis. A number of scoring systems have been proposed for this purpose, most of which are based on the presence and extent of inflammation in the interior of the paranasal sinuses. The Lund-McKay [21] scoring system is an objective method for the evaluation of opacification of the sinuses on tomograms that eliminates the occurrence of false positives or negatives. A clinical exam and computed tomography performed by an otolaryngologist allows a precise diagnosis of sinus disease, which can present in a similar manner without necessarily being maxillary sinusitis. Moreover, a number of studies have demonstrated that cone-beam computed tomography (as employed in the present study) is a good imaging tool for the evaluation of sinus disease.

The prostheses supported by zygomatic implants have a special design due to the location and a more palatal emergence profile of the implants in position when compared to conventional implants. This situation can hinder the tongue position and hygiene of the prosthesis and interfere with function [14, 31]. Some studies [6, 16, 31] conducted an assessment of the level of patient satisfaction on the prosthesis supported by zygomatic implants, demonstrating good levels of acceptance. Farzad et al. [16] evaluated the satisfaction of patients undergoing placement of zygomatic implants by Stella and Warner’s technique and compared with a group rehabilitated with full fixed prosthesis without zygomatic implants also using VAS.

No statistically significant differences have been found considering the different aspects analyzed, except with respect to aesthetics. In our study, there were significant differences in both overall satisfaction as the specific items assessed showed better results in total fixed prosthesis without zygomatic implants, although group I, represented by the PTF with zygomatic implants, has achieved good averages, except in the ease of entry for cleaning the prosthesis [15, 20, 31].

Farzad et al. [16] in their assessment of patient satisfaction after rehabilitation did not describe changes in speech. However, in our study three patients rehabilitated with zygomatic implants complained of difficulty in the ability to speak, especially when pronouncing words with the letter “s”. Nakai et al. [31] also reported the presence of patients complaining about speech, one patient complained for 3 months and the other one for 2 weeks, both after installation of the prosthesis. Brånemark et al. [14] and Nakai et al. [31] correlated problems in speaking with the design of the installed prostheses in patients with zygomatic implants which differs from those who are treated with conventional implants with or without the need for grafting.

Hirsch et al. [6] evaluated the satisfaction at the time of insertion of fixed prostheses and after 1 year of follow-up in 76 patients treated with 124 zygomatic implants. Complete satisfaction was observed with the cosmetic and functional results in 80 % of these patients, in both time frame analyzed. Farzad et al. [16] also used a VAS to assess patients’ response to treatment with zygomatic implants, describing difficult to chew and less satisfaction with respect to aesthetics, that can be related to the subjectivity of the analyses. In our study, both groups of patients presented good results with respect to aesthetics and function, but the conventional implant group showed the highest rate for both questions. For the group with zygomatic implants, two patients in the cosmetic item reported that the prosthesis did not show the expected results, which may have been influenced by the individual’s subjective opinion.

Analyzing masticatory function and stability, the group without zygomatic implants showed better results that can be explained by the fact that 85.7 % of the total antagonists are fixed implant prostheses or natural dentition, against 57.14 % in group I.

## Conclusions

The findings of our study showed that the technique of Stella and Warner allows the installation of zygomatic implant with high predictability, having achieved a high survival rate, and the absence of maxillary sinusitis, with a good level of satisfaction. These findings are important to confirm the efficacy and clinical applicability of the technique and demonstrate the low complication rate. However, the development of new studies with longer follow-ups and a larger number of patients involved in the sample is necessary to enhance the scientific evidence in this choice of treatment.

### Consent

Written informed consent was obtained from the patients for publication of this study and any accompanying images.

### Ethics approval

This retrospective cohort study was submitted and approved by the Hospital Universitário Onofre Lopes Research Ethics Committee, receiving the registration number 137/201.

### Ethical responsibilities

We declare that the authors of this study respect the ethical responsibilities which included the standards of this magazine, maintaining the confidentiality and privacy of patients, without identifying them in the text and images, and in agreement with the terms of the SpringerOpen Copyright and License Agreement, which we strongly recommend you read.
